# Enhanced registration of ultrasound volumes by segmentation of resection cavity in neurosurgical procedures

**DOI:** 10.1007/s11548-020-02273-1

**Published:** 2020-10-07

**Authors:** Luca Canalini, Jan Klein, Dorothea Miller, Ron Kikinis

**Affiliations:** 1 Fraunhofer MEVIS, Institute for Digital Medicine, Bremen, Germany; 2 Medical Imaging Computing, University of Bremen, Bremen, Germany; 3 Department of Neurosurgery, University Hospital Knappschaftskrankenhaus, Bochum, Germany; 4 Surgical Planning Laboratory, Brigham and Women’s Hospital, Harvard Medical School, Boston, USA

**Keywords:** Ultrasound, Image-guided surgery, Resection cavity, Deep learning, Neurosurgery

## Abstract

**Purpose:**

Neurosurgeons can have a better understanding of surgical procedures by comparing ultrasound images obtained at different phases of the tumor resection. However, establishing a direct mapping between subsequent acquisitions is challenging due to the anatomical changes happening during surgery. We propose here a method to improve the registration of ultrasound volumes, by excluding the resection cavity from the registration process.

**Methods:**

The first step of our approach includes the automatic segmentation of the resection cavities in ultrasound volumes, acquired during and after resection. We used a convolution neural network inspired by the 3D U-Net. Then, subsequent ultrasound volumes are registered by excluding the contribution of resection cavity.

**Results:**

Regarding the segmentation of the resection cavity, the proposed method achieved a mean DICE index of 0.84 on 27 volumes. Concerning the registration of the subsequent ultrasound acquisitions, we reduced the mTRE of the volumes acquired before and during resection from 3.49 to 1.22 mm. For the set of volumes acquired before and after removal, the mTRE improved from 3.55 to 1.21 mm.

**Conclusions:**

We proposed an innovative registration algorithm to compensate the brain shift affecting ultrasound volumes obtained at subsequent phases of neurosurgical procedures. To the best of our knowledge, our method is the first to exclude automatically segmented resection cavities in the registration of ultrasound volumes in neurosurgery.

## Introduction

In the neurosurgical planning for tumor resection, preoperative magnetic resonance imaging (MRI) data are usually acquired [1, 2]. Subsequently, neuronavigation systems are utilized to make these images available during the tumor resection. Through a rigid transformation computed between the surgical scene and the MRI data, neurosurgeons are able to map any intracranial position to the preoperative data. This is beneficial for the surgery outcome, since it decreases the risk of tumor residuals and increases the survival rate of the operated patients. However, image-guided surgery based only on preoperative data has some limitations [3]. In the early stages of the procedure, inaccuracies in the neurosurgical setting can degrade the rigid registration computed by the neuronavigation systems. Moreover, during the ongoing procedure, several anatomical modifications take place and the observed surgical scene modifies compared to the preoperative data. In the early stages of the surgery, the opening of the dura mater is responsible for the leakage of cerebrospinal fluid that heavily modifies the brain structure. Additionally, the resection of the tumor leads to other anatomical modifications, with no counterpart in the preoperative data. All these effects combined together are denoted as brain shift [3]. This phenomenon impedes a correct mapping between preoperative data and surgical scene. Consequently, the probability of missing pathological tissue in the resection increases, reducing the survival rates of the operated patients [4, 5].

To compensate the brain shift, intra-operative images can be acquired to provide an update of the resection scene [6]. The most common intraoperative solutions are given by MRI and ultrasound (US) modality. Intraoperative MRI (iMRI) data give a good image contrast between healthy and pathological tissues [7, 8], but it has the disadvantages to be expensive, to require special adaptation in the operating room (OR) and to be relatively long to be acquired. A valid alternative is given by intraoperative US (iUS), which is inexpensive, fast and practical to obtain [9, 10]. However, the understanding of the US data can be challenging [11], in particular if compared to the image quality obtained by MRI modality. To overcome this problem, neuronavigation systems can provide a direct mapping between preoperative MRI and iUS. By observing the same structures in two different imaging modalities, a better understanding of the tissues is also possible.

Additionally, a source of artifacts negatively affecting iUS is related to the resection cavity (RC), which appears in the ongoing procedure [11]. To perform US acquisitions after a first resection, a saline water solution is used to fill the operative cavity. When the US probe is used, the attenuation of sound waves in tissue is higher than in the saline water solution used for coupling. Consequently, hyperechoic artifacts appear, especially at the bottom of the resection cavity. They negatively affect the interpretation of the images, since they can be wrongly seen as pathological tissue. Therefore, toward the end of the resection, it becomes extremely important to identify these artifacts. As a solution, US volumes can be obtained at different time points of the resection, without a delay in the surgical procedure [2]. By tracking the US probe, neuronavigation systems compute a registration of the US volumes acquired at different phases of the resection. Then, the US data obtained at the end of the surgery can be compared with the early stage acquisitions [11], in which the artifacts related to the resection did not appear yet. Thus, the image interpretation becomes easier. Nonetheless, the direct comparison between subsequent phases is challenging due to the brain shift, which can only be compensated by a non-rigid solution [1, 12]. The registration provided by the neuronavigation systems is often not accurate, since it does not take into account anatomical modifications [1]. Therefore, this task is an open issue and a solution is still needed.

**Fig. 1:**
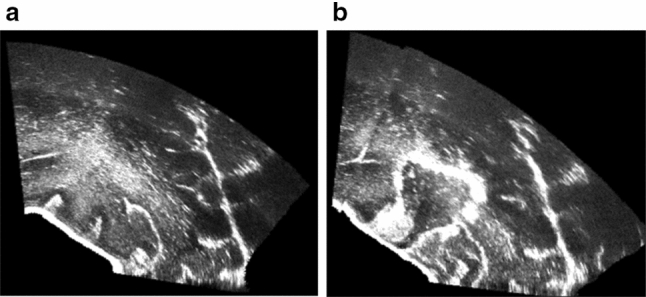
Ultrasound images acquired before (**a**) and after (**b**) resection. During the ongoing resection, the tumor is gradually removed and the cavity of the resection appears. The resection cavity is usually filled with saline water, which appears usually hypoechogenic in US acquisitions. The extension of the resection is usually recognizable by a hyperechogenic contour. By comparing images **a**, **b**, we can notice that other anatomical elements remain visible, even if deformed due to brain shift effect. On the contrary, the resection cavity has no specific counterpart in the initial acquisitions. In the process of registering the two images, it would be beneficial to exclude the non-corresponding elements of the resection cavity and rely only on the structures which remain visible among subsequent acquisitions

The registration of US volumes acquired at different resection stages is challenging, since the brain undergoes anatomical modifications, such as the emerging resection cavity, which have no counterpart in the data acquired at the beginning of the surgery (Fig. 1). A few registration solutions, which take into account the missing correspondences between the different acquisitions, have been already proposed. In [13], the authors suggested a non-rigid registration algorithm that models the deformation field with free-form cubic B-splines. In the cost function, the similarity metric is based on the normalized cross-correlation (NCC). They also introduced a method to suppress the effect of non-corresponding regions between the pre- and post-resection ultrasound volumes. The outlier detection is based on the standard deviation of the NCC gradients. The same approach has been applied in [14]. The authors in [15] advised an improvement compared to [13]. They proposed a symmetric deformation field and an efficient second-order minimization for a better convergence of the method. Moreover, an outlier detection to discard non-corresponding regions between volumes is proposed. Their approach starts from the one applied in [13] and adds an additional feature to improve the accuracy of outlier detection. Another solution considering also the resection cavity has been proposed by [16] to tackle the registration of pre- and intra-operative MRI images. Their framework is based on the Demons algorithm using an anisotropic diffusion smoother. The resected tissues in intra-operative data are detected with a level set method and then integrated into the smoother as a diffusion sink.

Furthermore, the effect of the outliers can be reduced by feature-based methods, limiting the registration only to corresponding elements. In [17], the authors proposed a feature-based registration approach where corresponding images features are computed in US pairs. Afterward, they used them to estimate a dense mapping through the images. The authors utilized several datasets to test the validity of their approach. Besides, the authors in [18] proposed a segmentation-based method. In the first step of their approach, they introduced an automatic segmentation of corresponding elements (sulci and falx cerebri) in subsequent US acquisitions. Then, their registration solution is only based on the automatically generated masks. In this way, they discarded the non-corresponding elements by focusing on structures available in the different acquisitions. A similar approach has been proposed in [19], where the authors applied a distance transform on the generated segmentation of the sulci and falx cerebri. Then, the transformed masks were registered.

We here propose a solution to register subsequent US volumes by discarding the non-corresponding elements from the registration process. In our approach, a 3D convolution neural network is utilized to segment the cavities generated with the tumor removal. Then, in the following step, the generated masks are used to discard the contribution of the resection cavity from the registration process. We expect the proposed registration approach to achieve better results than the same method not excluding the resection cavity. Regarding the segmentation step, to the best of our knowledge only the authors in [20, 21] proposed a solution for this task. In [21], they described a method based on a 2D U-Net to segment the resection cavity in US volumes. Besides, in [20] they also demonstrated that the 3D architecture achieves better results than a 2D approach. The mean time required from their 3D neural network to process a volume is around 5 min, making the application of their method not feasible in clinical scenario. For both their methods, the authors used a private ground truth to train their neural network.

## Material and methods

### Datasets

We utilized two publicly available datasets containing US acquisitions obtained at different stages of tumor resection [1, 12]. In both datasets, manually annotated landmarks are given for testing registration algorithms. The *RESECT* dataset [1] includes US volumes acquired at three different stages of the resection. As mentioned in the original publication, we indicate with *before resection* the acquisition at the beginning of the neurosurgery, when the dura mater is typically intact. After an initial resection is performed, a further acquisition is performed, and we refer to it as *during resection.* At the end of the resection, neurosurgeons verify if any pathological tissue is left, and the performed acquisitions are referred to as *after removal*. To have more details about the initial mean target registration error (mTRE) and the number of landmarks per each pair of volumes, please refer to Tables 2 and 3. The *BITE* dataset [12] was released before the RESECT one and contains volumes acquired before and after resection. Ten landmarks are provided per each pair of volumes, and initial mTRE is provided in Table 4. The quality of the images of BITE dataset is lower compared to the more recent RESECT dataset, as observed by [1]. Moreover, as observed by [20], the acquisitions protocols of this dataset differ from the one in the RESECT data (more details in Fig. 7).

### Manual annotations

**Fig. 2:**
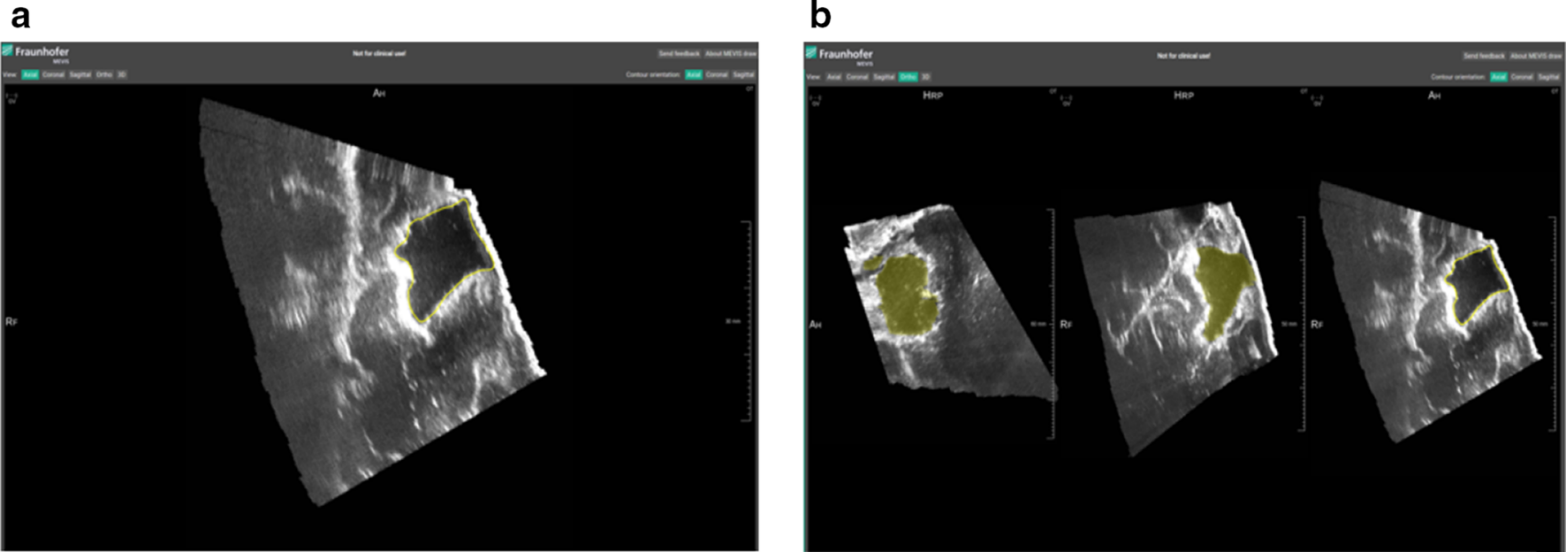
Annotation tool. The contour of the structure of interest is drawn on a specific view (**a**). After having drawn contours on a limited number of images, an interpolation automatically computes the contours on the remaining slices. In the process of segmenting the element of interest on a specific projection (**a**), the corresponding contour can be visualized in the other two views (**b**)

**Fig. 3:**
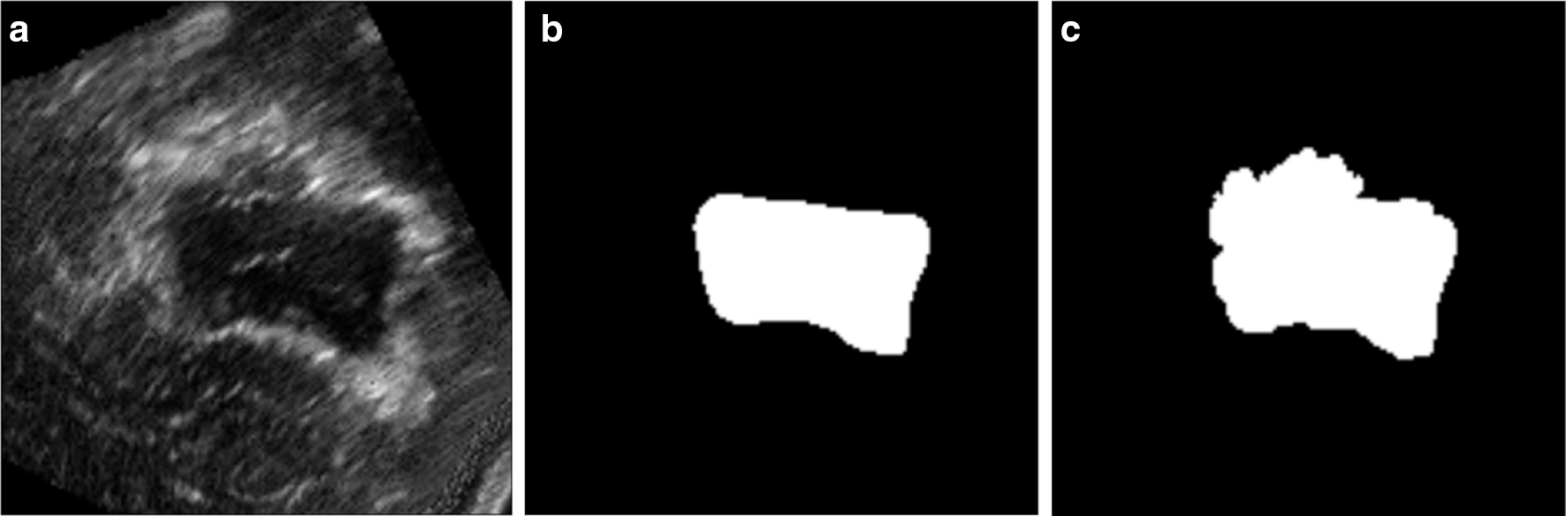
Manual segmentation. Two raters annotated the masks of interest. As an example, a the image of an original intensity volume. In **b** (first rater) and **c** (second rater), the two different versions of the manual segmentation of the corresponding resection cavity are provided

No ground truth for resection cavity segmentation is provided in the aforementioned datasets. Thus, we decided to manually annotate 27 volumes of the RESECT dataset, acquired during and after resection. To manually annotate the resection cavities in the US volumes, we utilized MEVIS Draw^1^ (see Fig. 2). With this tool, the volume of interest can be visualized in three main projections and the user can choose the more appropriate one for performing the manual segmentation (Fig. 2a). If the annotation is executed on non-adjacent slices, an interpolation automatically fills in contour on slices not yet annotated. This reduces the time needed for the annotation procedure, making 3D segmentation very efficient. Furthermore, the user has always the chance to observe the manual annotation in three different views (b), in which the drawn contours are projected. In case a modification is needed, the user can modify the manual annotation in any of the three views. Then, the interpolation is processed again. Two raters (L.C. and D.M.) annotated the resection cavities in the volumes of RESECT specified in Table 1 (Fig. 3). The author L.C. has two years of experience with ultrasound data, and the co-author D.M. is a neurosurgeon with a long experience in the use of US for tumor resection [18]. The masks generated by the intersection of the two manual segmentations are available at the following link (https://owncloud.fraunhofer.de/index.php/s/sv5je6Rkm4uYr7s).

**Table 1:** DICE coefficient for the segmentation of the resection cavity

Training set																	
Volume	2 a	2 d	3 a	4 a	7 a	7 d	12 d	15 a	16 a	16 d	17 a	18 a	19 a	21 a	24 a	25 d	27 a
DICE	0.91	0.91	0.85	0.88	0.89	0.7	0.88	0.82	0.88	0.82	0.91	0.90	0.95	0.95	0.86	0.87	0.92

Validation set						Test set				
Volume	1 a	14 a	17 d	19 d	21 d	6 a	6 d	12 a	18 d	25 a
DICE	0.76	0.88	0.75	0.75	0.71	0.88	0.88	0.88	0.26	0.86

The first part of the table is related to the training set, whereas the second one for the validation and test sets. The second row of each table indicates the RESECT US volumes of interest: Each volume is indicated by a number, specifying its related case in the dataset, followed by a letter. The letter indicates if the volume of interest is related to the acquisition performed during (d) or after (a) resection. For example, 4 a is used for the volume belonging to Case 4 acquired after resection. The third row indicates the computed DICE indices

### Automatic segmentation

We used the clinically revised masks as a ground truth to train a convolution neural network (CNN). CNNs are a class of artificial neural networks that automatically and adaptively learn spatial hierarchies of features from images in order to solve specific tasks (classification, segmentation, etc.) [22]. CNN is generally composed by three types of blocks: convolution, pooling and fully connected layers. In a training phase, the generated outputs are compared with the ground truth and a loss function is defined to measure the performance of the CNN. Thanks to a backpropagation algorithm, the trainable parameters are iteratively changed in order to minimize the loss function and therefore to reduce the difference between generated outputs and ground truth. A stochastic gradient descent optimization algorithm is usually used. For the segmentation task, we utilized a neural network whose architecture is based on the 3D U-net [23]. It consists of an analysis path to capture anatomical context and a symmetric synthesis path to achieve a precise localization the structures of interest. An image is given in input to the contracting (analysis) path. Here, each layer has two convolutions followed by an activation function and a max pooling: Feature maps are extracted from the image passing through the first part of the network. Then, in the expanding (synthesis) path, the image is upsized to the original size. Each layer consists of an upconvolution followed by two convolutions and activation function. In the synthesis path, at each level the upsized image is combined with the corresponding high-resolution features extracted in the contracting path. In output of the neural network, a volumetric mask is generated and an error between the generated mask and ground truth is computed.

With respect to the original implementation, some modifications were empirically made: The analysis and synthesis paths have three resolution steps and before each convolution layer of the upscaling path a dropout with a value of 0.3 is used in order to prevent the network from overfitting. Additionally, we utilized the Tversky loss function [24] which penalizes the false-negatives and thus advantages the over-segmentation of the foreground structures. We decided to train with this loss function because it has been shown to achieve good results in case of an unbalanced dataset. The training of the CNN is conducted with patches of size (48, 48, 48), a padding of (20, 20, 20) and a batch size of 5 samples. The learning rate was set to 0.0005. Additionally, we divided the annotated volumes into three groups (more details in Table 1). The first set (training set) includes 17 volumes (60% of the total volumes) and is used to train the neural network. The second one is disjoint from the training set and includes 5 volumes (20% of the dataset): It is utilized to validate the trained algorithm on volumes different from the one used for training. Then, after the best model has been saved, the network is applied on a test set (5 volumes) not used during validation and training. To evaluate the effects of differences in the segmentation on the registration results, two 3D U-Net models are trained, each using the annotation manually segmented by a different rater.

As commonly happens in medical images, in our dataset the positive voxels of the foreground are in minor quantity than the background ones, and this may slow the learning process of the neural network. To speed the training, we decided to alter the composition of the patches used to train the neural network. Thus, during the training and validation processes, we decided to feed the network with 20% of patches including only the background. The rest of the 80% includes patches containing at least a pixel with the foreground label. In our experiments, the Tversky loss had often the effect of producing an over-segmentation of structures of interest. The resection cavity is usually a closed isolated structure, with no dispersed elements. Therefore, we applied a connected component analysis on the automatically generated masks, to keep only the biggest segmented structure, corresponding to the resection cavity.

### Registration

In the proposed solution, the volume acquired before resection represents the template (moving) image which is deformed to match the reference (fixed) one, respectively, the data acquired during or after resection. The proposed solution is based on [25], where the registration of two volumes is treated as an iterative optimization algorithm. In this scenario, the correct registration of the two images corresponds to the global minimum of a discretized objective function: This includes a distance measure, determining the similarity between the deformed template and the reference image, and a regularizer, which penalizes undesired transformations. In addition to the moving and fixed images, the proposed method uses an additional input (mask). The parts excluded from the masks are usually those not in common in both acquisitions. Thus, the contribution to the distance measure is limited to the areas for which the segmentation is available.

In our solution, we use the quasi-Newton l-BGFS [26] to guide the optimization process and the normalized gradient field (NGF) as the distance measure. The stopping criteria for the optimization process are empirically decided: The minimal progress, the minimal gradient and the relative one, the minimum step length were set equal to 0.001, and the maximum number of iterations is equal to 100. The algorithm takes in input two original intensity volumes. Besides, the automatically generated masks are provided as third input. The area of the resection cavity is excluded from the computation of the distance measure: This represents the only element not in common between the two input images, and its exclusion may improve the registration output. Moreover, as suggested by [27] and [28], the background outside the US beam is also discarded. The proposed approach is initialized by a rigid registration, followed by a deformable approach. In the first step, the volumes are directly registered at one fixed level coarser than the original scale, in order to improve the speed of the algorithm. A transformation matrix is obtained and then utilized for initializing the deformable registration. In the second step, we utilized the curvature regularizer to limit the range of possible transformations [25]. To avoid local minima in the optimization and to speed the computation, the volumes are registered from a third coarse level, in which computations are cheap, until the fine level. Besides, to estimate the effects of the inter-variation in the segmentation on the registration results, we test the method with the two versions of the masks generated by the two 3D U-Net models. We would like to check how much the differences in the masks to be excluded affect the registration results.

In our experiments, we also tested a solution not excluding the resection cavity from the registration.

## Results

### Segmentation

**Fig. 4:**
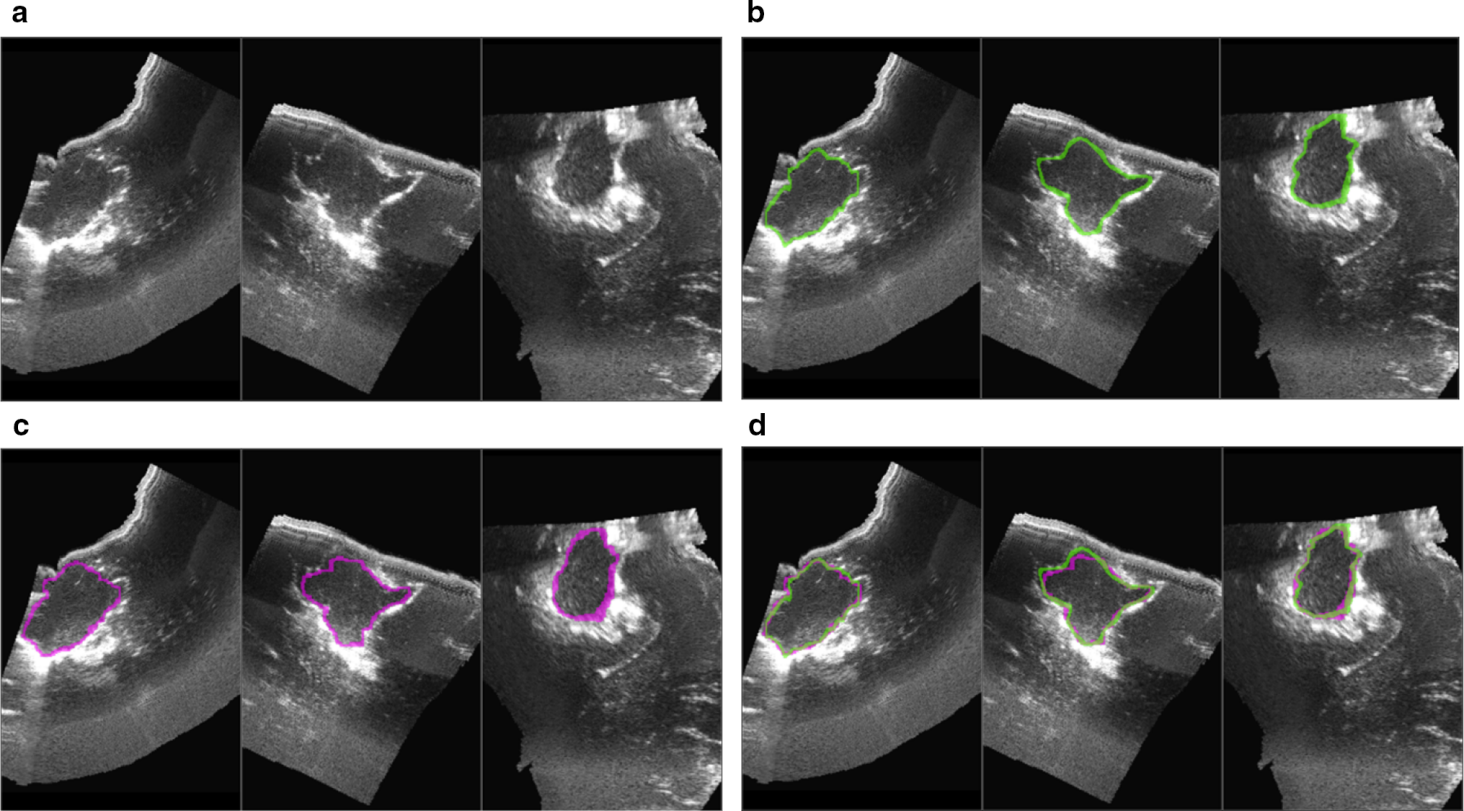
Example of automatic segmentation in a volume acquired during resection. **a** The original intensity volume, on which a manual annotation has been drawn (in green) (**b**). The automatically computed mask is visible in **c** (in pink), whereas a direct comparison between the ground truth and the generated masks is given (**d**)

**Fig. 5:**
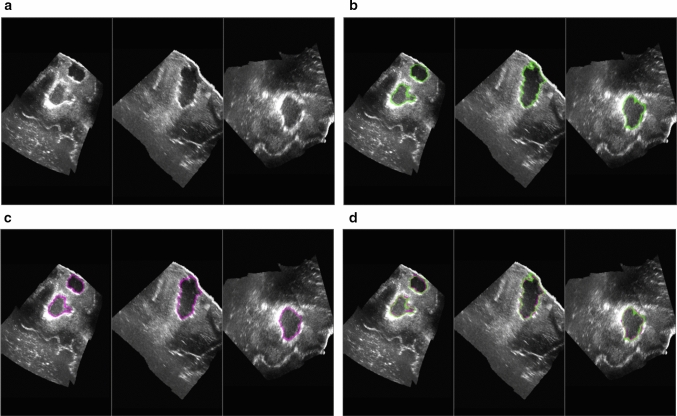
Example of automatic segmentation in a volume acquired after resection. **a** The original intensity volumes, on which a manual annotation has been drawn (in green) (**b**). The automatically computed mask is visible in **c** (in pink), whereas a direct comparison between the ground truth and the generated masks is given (**d**)

**Fig. 6:**
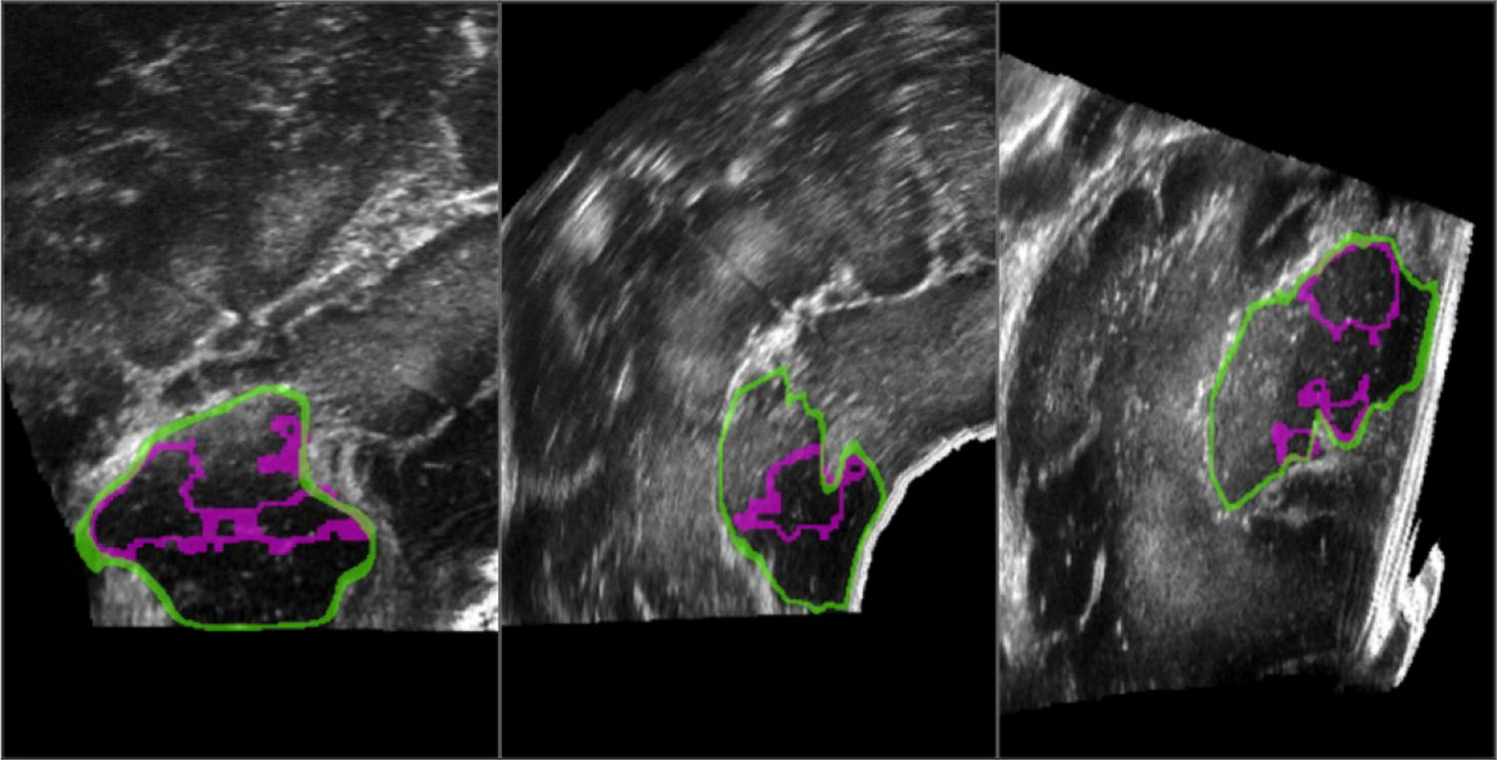
Worst-case segmentation. This figure is a visual inspection of the volume 18 during resection for which we obtained the worst DICE index. The green contour is related to the ground truth, whereas the purple one to the automatic segmentation. The generated mask is smaller compared to the manual annotation

Table 1 shows the DICE coefficients for the segmentation of the resection cavity for the 3D U-Net model obtaining the best mean value. Overall, the mean DICE coefficient is 0.84. Visual results are available in Figs. 4 and 5, respectively, for the case 2 acquired during resection and for the case 27 acquired after resection. Each figure shows the related volume in three projections. To maintain a good tradeoff between the visibility of the surrounding anatomical structures and visualization of the mask of the resection cavity, we decided to highlight the element of interest with a border in green (ground truth) and purple (automatic segmentation). Figure 6 shows the segmentation results for the volume 18 acquired during resection (18 d in Table 1), for which the worst DICE coefficient (0.32) has been obtained. In the figure, we show the overlay between ground truth and automatic segmentation.

**Fig. 7:**
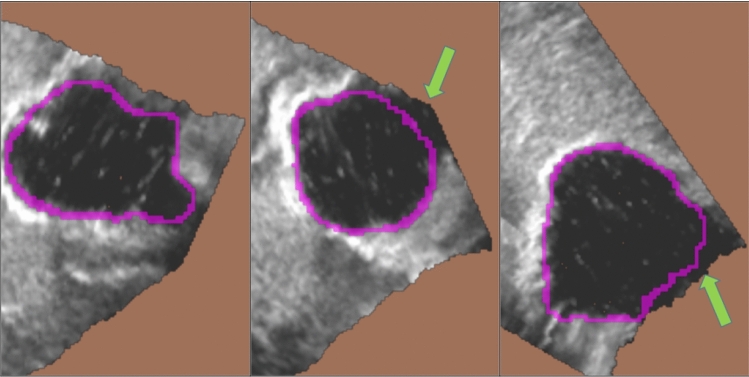
Segmentation results on resection cavity with an open border. This figure shows the result of the automatic segmentation (purple border) on a BITE dataset volume. The background surrounding the US cone is colored in orange. By looking at the positions pointed by the green arrows, we can see how a side of the resection cavity is not surrounded by a hyperintense contour: The US probe is directly inserted in the RC, and this side of the cavity has no enhanced border. The acquisition procedure of the volumes included in BITE dataset differs from the RESECT one, in which the resection cavities is always surrounded by a hyperintense contour. Our segmentation algorithm correctly segments the structure of interest when it is surrounded by a hyperintense element. However, it under-segments the part of the cavity where no hyperintense border is available (the hypointense parts pointed by the arrows should be included within the purple border)

After having trained the neural network on the RESECT dataset volumes, we applied it to volumes after resection of BITE dataset. As observed by [20], the acquisitions methodologies and the quality of the volumes are different between the two datasets. The cavity is sometimes partially visible in BITE dataset, whereas on the contrary in the RESECT volumes it is always completely observable and usually surrounded by a bright border. In Fig. 7, we show an example of a BITE case segmented by our methodology.

The proposed 3D U-Net requires a mean time of 4.86 s to generate the mask of interest.

### Registration

After having registered the volumes, the deformation field is applied to the related landmarks. The mTRE obtained excluding the masks generated by the algorithm trained with the segmentation of first rater is 1.25 mm and 1.24 mm (before-during and before-after registration, respectively), whereas the results by the method discarding the resection cavity segmented with the 3D U-Net designed with the masks of the second rater are 1.22 mm and 1.21 mm. In Tables 2 and 3, we report the detailed results of the second method. Table 2 is related to the volumes before and during resection, whereas Table 3 refers to those before and after tumor removal. In both the tables, the fourth column is related to the results obtained by our solution without the exclusion the resection cavity, whereas in the last one we report the registration results achieved discarding it. For the volumes acquired during and before resection, we achieved a mTRE of 1.22 mm by excluding the resection cavity and 2.57 mm without excluding it. Instead, the corresponding results for the volumes acquired after and before removal are of 1.21 mm and 3.53 mm. For a better comparison with previously proposed methods, each table also contains a second section in which the mTRE achieved by other methods is listed.

**Table 2:** Registration results for volumes acquired before and during resection (RESECT dataset)

Patient ID	# Of landmarks	Mean initial distance (mm)	Without masking	With masking
1	34	2.32 (1.49–3.29)	2.32 (1.49–3.29)	0.64 (0.20–1.72)
2	16	3.10 (1.79–5.19)	1.57 (0.56–4.07)	1.50 (0.32–4.20)
3	17	1.93 (0.67–3.02)	0.86 (0.18–2.14)	0.77 (0.29–1.39)
4	19	4.00 (3.03–5.22)	0.82 (0.22–2.38)	0.80 (0.20–2.40)
6	21	5.19 (2.60–7.18)	7.17 (0.54–12.58)	5.17(0.25–10.36)
7	22	4.69 (0.94–8.16)	1.95 (0.47–5.82)	1.98 (0.32–6.17)
12	24	3.39 (1.74–4.81)	2.01 (0.08–8.66)	0.84 (0.16–2.02)
14	22	0.71 (0.42–1.59)	0.49 (0.11–1.19)	0.41 (0.09–1.11)
15	21	2.04 (0.85–2.84)	6.37 (2.03–11.15)	0.60 (0.16–1.29)
16	19	3.19 (1.22–4.53)	11.23(9.20–13.26)	1.26 (0.06–3.40)
17	17	6.32 (4.65–8.07)	1.76 (0.33–4.70)	1.49 (0.25–3.69)
18	23	5.06 (1.55–7.44)	1.25 (0.26–3.98)	1.18 (0.34–3.76)
19	21	2.06 (0.42–3.40)	2.06 (0.20–6.98)	0.96 (0.12–2.76)
21	18	5.10 (3.37–5.94)	4.54 (0.51–9.63)	1.11 (0.18–3.91)
24	21	1.76 (1.16–2.65)	0.96 (0.22–2.72)	0.67 (0.17–1.44)
25	20	3.60 (2.19–5.02)	0.55 (0.15–1.61)	0.55 (0.18–1.61)
27	16	4.93 (3.61–7.01)	1.06 (0.05–2.92)	0.87 (0.15–2.19)
Mean value + SD	20.0 ± 4.8	3.49 ± 1.55	2.57 ± 2.93	1.22 ± 1.20
Other methods				Mean value
[18]				1.36

The table shows the registration results (mTRE registration errors in mm, the range (min–max) of the distances in parenthesis, and the standard deviation) obtained by our solution. The different cases (first column) come with landmarks, which are specified in the second column. In the third column, we show the initial registration. We compare the results obtained without (fourth column) and with applying (fifth column) the exclusion of the resection cavity previously segmented. A second section (other methods) of the table shows the mean TRE obtained by previously proposed methods

**Table 3:** Registration results for volumes acquired before and after resection (RESECT dataset)

Patient ID	# Of landmarks	Mean initial distance (mm)	Without masking	With masking
1	13	5.80 (3.62–7.22)	4.88 (1.02–7.44)	1.03 (0.18–3.23)
2	10	3.65 (1.71–6.72)	3.28 (1.46–6.16)	3.90 (2.36–5.88)
3	11	2.91 (1.53–4.30)	4.47 (1.68–6.42)	1.15 (0.34–1.93)
4	12	2.22 (1.25–2.94)	2.57 (0.38–4.84)	0.61 (0.13–1.17)
6	11	2.12 (0.75–3.82)	2.38 (0.19–6.01)	1.41 (0.26–4.70)
7	18	3.62 (1.19–5.93)	3.41 (0.52–5.62)	2.03 (0.13–4.88)
12	11	3.97 (2.58–6.35)	7.85 (5.77–11.03)	0.79 (0.32–1.92)
14	17	0.63 (0.17–1.76)	0.50 (0.19–1.04)	0.46 (0.15–0.98)
15	15	1.63 (0.62–2.69)	0.60 (0.21–1.48)	0.58 (0.19–1.25)
16	17	3.13 (0.82–5.41)	6.04 (3.51–8.84)	0.92 (0.28–2.27)
17	11	5.71 (4.25–8.03)	3.33 (0.90–6.17)	1.10 (0.23–4.89)
18	13	5.29 (2.94–9.26)	4.04 (0.69–6.63)	1.13 (0.37–3.13)
19	13	2.05 (0.43–3.24)	2.53 (0.50–8.60)	1.10 (0.41–2.40)
21	9	3.35 (2.34–5.64)	1.99 (0.52–3.47)	1.80 (0.84–3.39)
24	14	2.61 (1.96–3.41)	4.80 (3.17–7.12)	0.87 (0.35–2.42)
25	12	7.61 (6.40–10.25)	6.73 (3.29–9.20)	1.21 (0.15–5.65)
27	12	3.98 (3.09–4.82)	0.60 (0.17–1.79)	0.53 (0.14–0.90)
Mean value + SD	12.9 ± 2.6	3.55 ± 1.76	3.53 ± 2.12	1.21 ± 0.66
Other methods				Mean value
[17]				1.49
[18]				2.05
[19]				1.27
[29]				1.92

The table shows the registration results (mTRE registration errors in mm, the range (min–max) of the distances in parenthesis, and the standard deviation) obtained by our solution. The different cases (first column) come with landmarks, which are specified in the second column. In the third column, we show the initial registration. We compare the results obtained without (fourth column) and with applying (fifth column) the exclusion of the resection cavity previously segmented. A second section (other methods) of the table shows the mean TRE obtained by previously proposed methods

To determine whether the two proposed methods (with and without masking) show statistically significant difference, we conducted a statistical test. The data are not normally distributed, and thus, we decided to use the nonparametric Wilcoxon signed rank test. It tests the null hypothesis that two related paired samples (the results of the two algorithms) come from the same distribution. For both the studies (before-during and before-after), we verified that the null hypothesis cannot be accepted (*p* value < 0.001), meaning that there is statistical difference between the two methods. Besides, we conducted the same statistical analysis for the two registration methods using the masks generated by the two different 3D U-Net models. For both the registration tasks, the test fails to reject the null hypothesis (*p* value > 0.6).

Moreover, we tested our final solution also on the BITE dataset. The related results are available in Table 4.

**Table 4:** Registration results for volumes acquired before and after resection (the BITE dataset)

Patient ID	Mean initial distance (mm)	With masking
2	2.30 (0.57–5.42)	1.70 (0.65–4.11)
3	3.40 (0.0–5.09)	1.49 (0.25–3.91)
4	4.60 (2.96–5.88)	5.34 (3.09–8.89)
5	4.11 (2.58–5.52)	1.17 (0.41–2.32)
6	2.26 (1.36–3.10)	1.08 (0.38–2.38)
7	3.87 (2.60–5.07)	1.23 (0.54–2.09)
8	2.51 (0.67–3.93)	1.21 (0.45–2.32)
9	2.21 (1.00–4.59)	1.57 (0.26–4.22)
10	3.86 (0.98–6.68)	1.18 (0.44–2.26)
11	2.74 (0.44–8.22)	2.29 (0.20–7.49)
12	10.54 (7.85–13.04)	10.79 (7.68–13.34)
13	1.62 (1.33–2.21)	0.71 (0.25–1.76)
14	2.19 (0.59–3.99)	1.17 (0.34–3.10)
Mean value + SD	3.55 ± 2.28	2.38 ± 2.78
Other methods		Mean value
[13]		1.50
[14]		1.50
[15]		1.50
[17]		1.54
[18]		2.48

The table shows the registration results (mTRE registration errors in mm, the range (min–max) of the distances in parenthesis, and the standard deviation) obtained by our solution excluding of the resection cavity previously segmented. A second section (other methods) of the table shows the mean mTRE obtained by previously proposed approaches. The methods [13–15] were tested only on BITE dataset, whereas [17] also on the RESECT dataset, but only on the set of volumes acquired after resection

**Fig. 8:**
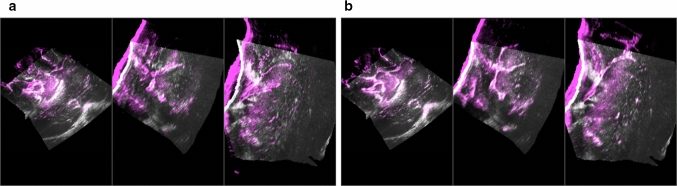
Registration of volumes acquired before and during resection. The two figures show the volumes before resection (in purple) overlaid on the volumes during resection (in gray). **a** The situation before applying our registration algorithm; **b** the overlay of the volumes after having deformed the moving image

**Fig. 9:**
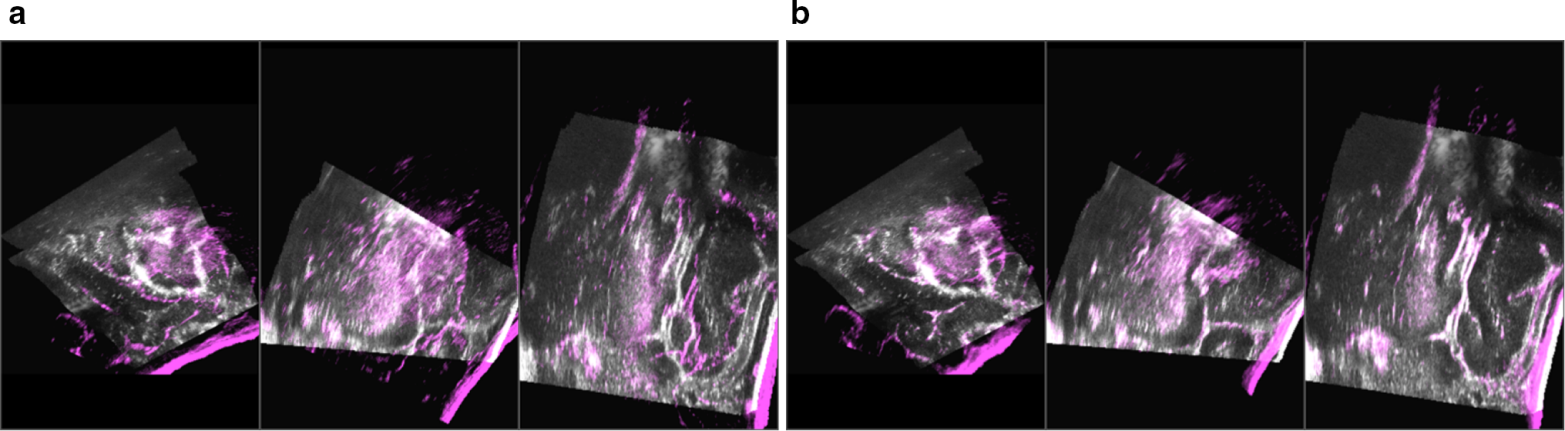
Registration of volumes acquired before and after resection. The two figures show the volumes before resection (in purple) overlaid on the volumes after resection (in gray). **a** The situation before applying our registration algorithm; **b** the overlay of the volumes after having deformed the moving image

The visual results for the registration task are shown in Figs. 8 and 9, displaying the data in three projections. The volumes before resection are colored in purple and are overlaid on the volumes during (Fig. 8) and after (Fig. 9) resection, shown in gray intensity. Each figure contains two sub-images displaying the overlay of the two volumes of interest, before (a) and after (b) having applied our registration algorithm. The difference between a and b is related to the volume before resection (in purple), which is deformed according to the deformation field computed by the registration algorithm.

The registration step requires a mean of 49.67 s to register the volumes of interest.

## Discussion

Regarding the segmentation approach, our solution is able to achieve a mean DICE index of 0.84 by comparing the automatically generated masks with the clinically validated ground truth. The visual results in Figs. 4 and 5 give evidence that the 3D U-Net achieves good results in segmenting the resection cavities in US volumes. The only exception is given by volume 18 during resection, for which our solution obtained the worst DICE index. This is confirmed also by visual inspection in Fig. 6: The resection cavity is under-segmented with respect to the manual annotation. The resection cavities are usually hypoechogenic structures contoured by a hyperechogenic border. However, for the case in Fig. 6, the resection cavity has intensity characteristics slightly different compared to the majority of the other volumes. Thus, a possible cause for this failure may be related to the intensity of the internal part of the resection cavity, which includes also a partial hyperechogenic area. Nevertheless, for the other volumes of test set, we obtained DICE values in line with the rest of the dataset (Table 1). Our algorithm has been also applied to the BITE volumes. Our automatic method is able to segment the volumes of interest, but it usually under-estimates the parts where a hyperintense border is missing. In this dataset, the US probe is usually inserted inside the resection cavity, whereas the volumes of the RESECT dataset are usually acquired from a position outside it. Consequentially, in the BITE dataset the hyper intense borders surrounding the cavity are not visible on the sides of the volume in which the acquisition probe has been interested (Fig. 7), whereas in the RESECT dataset the resection cavity is usually visible as a closed structure. The others algorithms proposed to segment the resection cavity used a privately defined ground truth to train their solutions. Therefore, a numerical comparison based on the DICE index is not possible. Regarding the time required to process a single volume, the solution proposed in [20] requires around 5 min. It may be due to (1) the deeper architecture of the neural network that they used, which requires more time to process a single volume and (2) the sampling method they chose, a sliding window approach with large patches. On the contrary, our algorithm is faster, requiring less than 5 s in the inference process.

Regarding the registration results, the proposed solution registers the volumes of interest by reducing the overall mTRE for all the sets of volumes taken into account. For the RESECT dataset, we are able to reduce the initial mTRE for all the volumes of both sets. Figures 8 and 9 also give impressions that the proposed registration algorithm produces a better alignment of the volumes, compared to the initial alignment. If compared the results obtained by using the masks generated by the two 3D U-Net models trained on different ground truth, we can observe that the results differ between each other: Changes in the segmentation have an impact on the registration outcome. However, these results also show that the differences in the masks are negligible on the registration results (less than 0.1 mm in terms of mTRE), as long as the segmentation includes the resection cavity. Moreover, from the numerical results obtained in the RESECT dataset, the exclusion of the resection cavity from the registration process (fifth column) provides better results than the case in which it is not excluded (fourth column). As expected, by discarding the non-corresponding elements from the registration process, the algorithm can focus on the elements in common and therefore obtain better results.

Compared to previous approaches, for both the sets of the RESECT dataset our algorithm achieves better results than the solutions compared in Tables 2 and 3. This is true for the algorithms proposed to register both sets [17, 18], but also for methods considering only the registration of volumes before-after resection [19, 29]. Additionally, our final method has been also tested on BITE dataset (Table 4), in which it is able to reduce the mTRE of each pairs of volume. In the comparison of our approach with previous solutions, the algorithms [13–15] were tested only on the BITE dataset: Even if they obtained slight better results than our solution, they lack generalization. On the contrary, our method has been tested on a larger set of volumes, providing a better generalization. Moreover, the overall mTRE is improved with respect to [18] (Table 4). However, the solution proposed by [17] achieves a better mTRE. Our results are mostly affected by the registration of volume 12, for which the initial error is only slightly reduced. The starting condition for the registration of this case is the worst of all the sets of volumes, and our method may be affected by the initial registration information provided by the optical tracking system.

Combining the time required by the segmentation of the resection cavity and the registrations step, the proposed algorithm is able to register two volumes in less than 55 s. This small delay to the neurosurgical procedure may be tolerable, especially if a better understanding of the surgical scene after the registration can be achieved.

## Conclusions

We presented here an automatic algorithm for 3D segmentation of resection cavities in US volumes, acquired in the neurosurgical procedures for tumor removal. For this specific method, we manually annotated a ground truth that has been made publicly available. Besides, we proposed a novel method to register US volumes acquired in neurosurgery context. In our solution, the resection cavities are excluded from the registration thanks to the automatic segmentation method, reducing the impact of non-corresponding elements in the computation of the distance measure. Our experiments show that the registration results are only slightly influenced by the differences in the masks, as long as they include the resection cavity to be excluded. On the contrary, we show that by omitting the exclusion of the resection cavities, a worsening of the results is obtained. To the best of our knowledge, it is the first time that the resection cavities are taken into account to improve the registration of US volumes in neurosurgical tumor resection. Moreover, the registration results obtained in the RESECT dataset are the lowest in comparison with the other methods in the literature (Tables 2, 3).

As future work, we plan to manually annotate the resection cavity in the volumes of BITE dataset, to propose a more generalized solution. Moreover, the registration method based on the exclusion of the resection cavity could be also utilized for the inter-modality registration of intraoperative US volumes and preoperative MRI.

## Footnotes

1 https://www.mevis.fraunhofer.de/en/research-and-technologies/image-and-data-analysis/mevis-draw.html.
